# Physics-informed neural network with transfer learning (TL-PINN) based on domain similarity measure for prediction of nuclear reactor transients

**DOI:** 10.1038/s41598-023-43325-1

**Published:** 2023-10-06

**Authors:** Konstantinos Prantikos, Stylianos Chatzidakis, Lefteri H. Tsoukalas, Alexander Heifetz

**Affiliations:** 1 School of Nuclear Engineering, Purdue University, West Lafayette, IN 47906 USA; 2 Nuclear Science and Engineering Division, Argonne National Laboratory, Lemont, IL 60439 USA

**Keywords:** Nuclear fusion and fission, Computer science

## Abstract

Nuclear reactor safety and efficiency can be enhanced through the development of accurate and fast methods for prediction of reactor transient (RT) states. Physics informed neural networks (PINNs) leverage deep learning methods to provide an alternative approach to RT modeling. Applications of PINNs in monitoring of RTs for operator support requires near real-time model performance. However, as with all machine learning models, development of a PINN involves time-consuming model training. Here, we show that a transfer learning (TL-PINN) approach achieves significant performance gain, as measured by reduction of the number of iterations for model training. Using point kinetic equations (PKEs) model with six neutron precursor groups, constructed with experimental parameters of the Purdue University Reactor One (PUR-1) research reactor, we generated different RTs with experimentally relevant range of variables. The RTs were characterized using Hausdorff and Fréchet distance. We have demonstrated that pre-training TL-PINN on one RT results in up to two orders of magnitude acceleration in prediction of a different RT. The mean error for conventional PINN and TL-PINN models prediction of neutron densities is smaller than 1%. We have developed a correlation between TL-PINN performance acceleration and similarity measure of RTs, which can be used as a guide for application of TL-PINNs.

## Introduction

Physical and engineering dynamical systems can be modeled with either ordinary differential equations (ODEs) or partial differential equations (PDEs), which can be solved analytically or numerically using finite difference method (FDM) and the finite element method (FEM). However, a new approach for solving differential equations (DE) has emerged recently that involves the use of deep learning neural networks (DNNs), which can be executed on special purpose hardware systems. DNNs were first proposed for solution of differential equations in 1998 by Lagaris^1^, which was later developed by Raissi^2,3^ and Karniadakis^4^ into physics-informed neural networks (PINNs). PINNs take advantage of the universal approximation feature of neural networks for solution of differential equations while offering a mesh- free approach without the domain discretization. Compared to traditional numerical solvers, such as FEM and FDM, PINNs utilize automatic differentiation^5,6^ (AD) which is an optimization technique. AD computes the derivatives using the chain rule for accumulation of values instead of relying on derivative symbolic expressions. PINNs integrate physical laws by incorporating governing ODEs/PDEs and initial conditions (IC) and boundary conditions (BC) into loss functions. This process establishes theoretical constraints and biases to supplement measurement data. PINNs can be applied to both supervised and unsupervised learning tasks^7–9^, as well as to forward and inverse problems^10^. PINNs training process requires substantially less data than for most deep learning methods because PINN performance is not directly related to the volume of training data. PINNs have attracted significant interest from researchers in a wide range of technical disciplines, including heat transfer^11,12^, structural dynamics^13,14^, fluid mechanics^15–18^, solid mechanics^19,20^, and nuclear reactor kinetics^21,22^.

In this work, we study the application of PINNs to monitoring nuclear reactor performance. Nuclear reactors are dynamic systems, in which reactor power can be regulated by the operator through a control mechanism. Development of advanced computational methods enhances the ability to model nuclear system transients, which equips reactor operator with tools to achieve better performance efficiency. The focus of our work is to expedite PINN training runtime using RTs modeling operation of Purdue University Reactor Number One (PUR-1) small research reactor. By using the system of point kinetic equations (PKEs), one can accurately model the operation of a small nuclear reactor, such as PUR-1. The PKEs consist of a system of stiff nonlinear ordinary differential equations that model time-dependent neutron flux density and several precursor groups. In this paper, to model reactor transients (RTs), we developed PKEs with the coefficients obtained from Monte Carlo N-Particle (MCNP) simulations of PUR-1.

Previous efforts utilized PINNs to solve PKEs and neutron diffusion models with promising results^23–25^. However, these approaches considered only hypothetical transients, not representative of experimental systems. For example, some authors solved simplified PKEs models that did not include neutron source for the start-up case, or did not consider six groups for the production of delayed neutron precursors. In our prior work^21,22^, we developed a solution of PKEs using conventional PINNs to model reactor start-up transient. In particular, we obtained PINN solution for six-group PKEs with a neutron source, and demonstrated interpolation and extrapolation capabilities of PINNs.

Using PINNs for reactor operator support applications is contingent on PINNs ability to execute in near-real time. In all machine learning based approaches, including PINNs, time-consuming model training and testing is required to achieve low error, i.e. in the range of 10^–5^ to 10^–4^, in model prediction. In this paper, we demonstrate that a transfer learning (TL-PINN) approach achieves significant performance gain, as measured by reduction of the number of iterations for model training. Transfer learning is the process of pre-training a neural network on similar data to enhance performance in a new task. Recent studies have investigated coupling of PINNs with transfer learning for several select applications^26–29^. To the best of our knowledge, the work in this paper is the first reported result on development of PINNs with transfer learning for monitoring a nuclear reactor.

In this paper, we developed a set of different RTs through computer simulations of PKEs for different reactivity insertion schedules. In all RTs, the range of neutron density values spans approximately nine orders of magnitude during time interval of several hundred seconds, which is consistent with the range of experimental values for typical PUR-1 performance. The RTs were characterized using Hausdorff distance, partial curve mapping (PCM), Fréchet distance, area between two curves, and dynamic time warping distance. First, we train PINN models, constructed using PKEs and fully connected feed forward neural network, to predict of neutron density and six groups of precursor densities. Next, PINN models pre-trained on one type of transients, are used to train and predict different types of RTs. Results show that TL-PINN approach provides order of magnitude performance acceleration compared to that of a conventional PINN model. Through numerical experiments, we have developed a correlation between TL-PINN performance gain and similarity measure of RTs, which can be used as a guide for application of TL-PINNs in a practical scenario.

The paper is organized as follows. The “Results” section discusses the details of numerical experiments involving prediction of different RTs with PINN and TL-PINN. The “Discussion” section summarizes performance benchmarking of PINN and TL-PINN. The “Methods” section describes the PKE model, the schematics of PUR-1, the architecture of PINNs, and similarity metrics for characterization of RTs.

## Results

**Figure 1:**
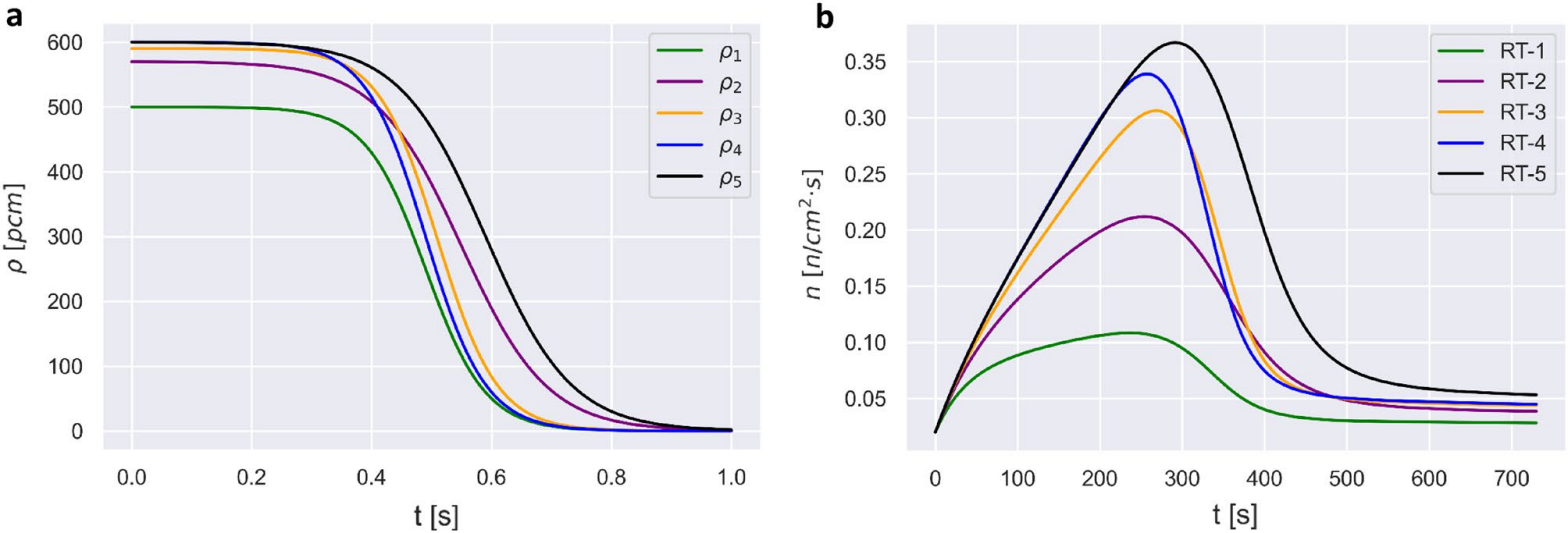
(**a**) Reactivity insertion schedules in normalized time domain ${\rho }_{1}\left(t\right)$ (green), ${\rho }_{2}\left(t\right)$ (purple), ${\rho }_{3}\left(t\right)$ (yellow), ${\rho }_{4}\left(t\right)$ (blue) and ${\rho }_{5}\left(t\right)$ (black), (**b**) Corresponding scaled neutron density $n\left(t\right)$ for RT-1 (green), RT-2 (purple), RT-3 (yellow), RT-4 (blue), and RT-5 (black).

Five nuclear reactor transients (RT-1, RT-2, RT-3, RT-4, and RT-5) were generated using ODE45 FDM solver of PKEs, which were constructed using experimental parameters of PUR-1. All five reactor transients consist of 742 s-long transients of neutron density $n\left(t\right)$ and delayed neutron precursor density concentration ${c}_{i}\left(t\right)$ for six groups. All RTs were generated via a positive reactivity insertion, as discussed in the Methods section. The range of values of $n\left(t\right)$ in all RTs is consistent with order of magnitude of typical experimental observations of PUR-1 operation. Transients of reactivity insertion schedules for five RTs are plotted on the normalized time domain $t\in \left[0, 1\right]$ s in Fig. 1a. Resulted transients of $n\left(t\right)$, scaled in the amplitude in the range of $\left[0, 0.4\right]$ n/cm^2^s , are shown in Fig. 1b.

For RT-1, $n(t)$ initially rises with approximately six-fold increase in the amplitude, reaching the maximum value at approximately 250 s (when reactivity begins to decrease), followed by decay to steady state. For RT-2, where the initial reactivity is larger by 70 pcm than that for RT-1, $n\left(t\right)$ increases by approximately a factor of 10, reaching the maximum value at approximately 280 s (when reactivity insertion begins to decrease), followed by decay to steady state. For RT-3, where the initial reactivity insertion is larger than that for RT-2 by 20 pcm, $n\left(t\right)$ increases by approximately a factor of 15, reaching the maximum value at approximately 270 s (when reactivity insertion begins to decrease), followed by decay to steady state. For RT-4, where the initial reactivity is larger than that of RT-3 by 10 pcm, $n\left(t\right)$ increases by approximately a factor of 17, reaching the maximum value at approximately 250 s (when reactivity insertion begins to decrease), followed by decay to steady state. For RT-5, where the initial reactivity insertion is the same as for RT-4, but with later reactivity insertion reduction, $n\left(t\right)$ increases by approximately a factor of 19, with the maximum value achieved at approximately 300 s (when reactivity insertion begins to decrease), followed by decay to steady state.

**Figure 2:**
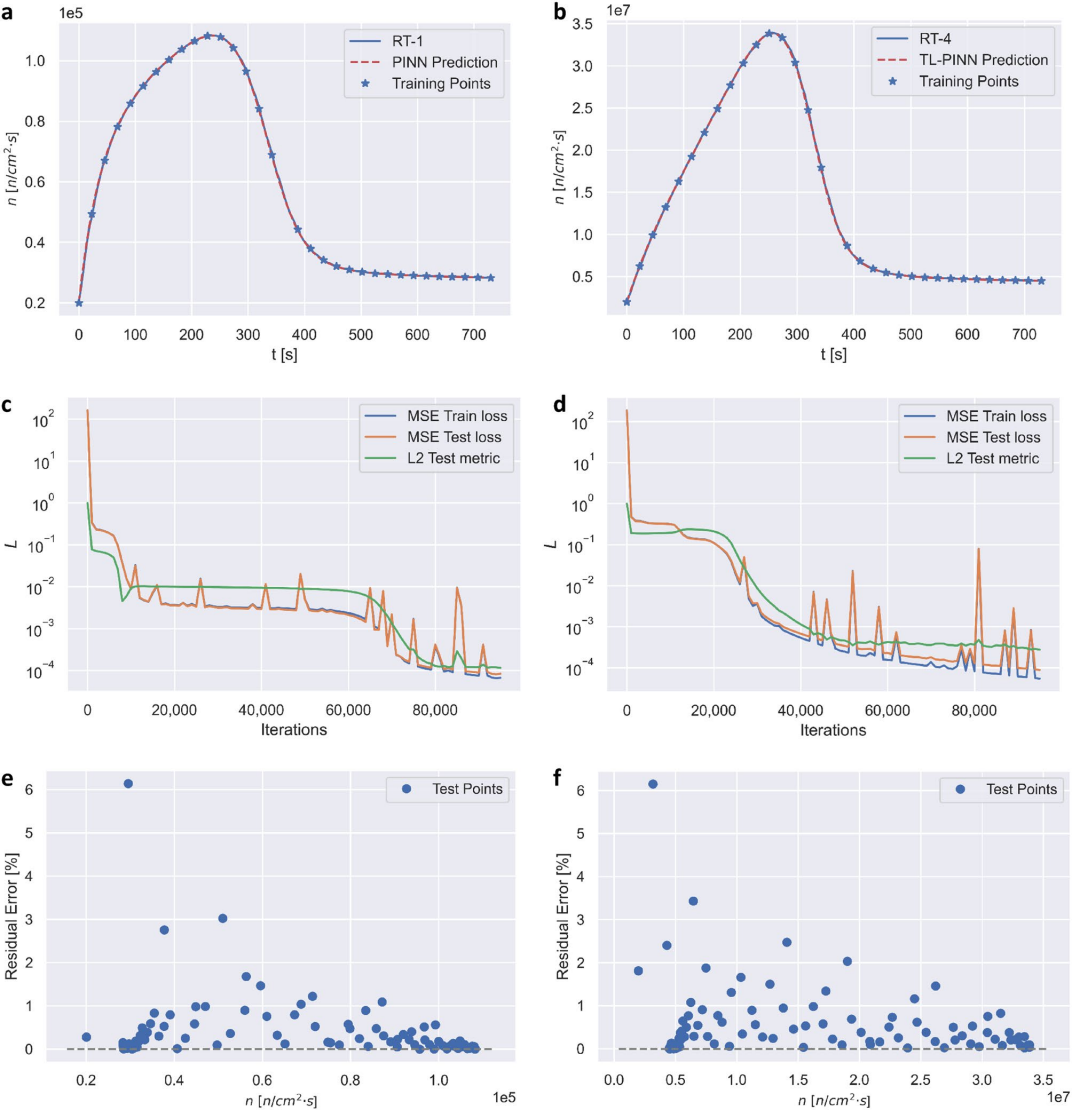
(**a**) Experiment 1: RT-1 $n(t)$ along with PINN training points and predictions. (**b**) Experiment 16: RT-4 $n(t)$ along with PINN training points and predictions. (**c**) Experiment 1: History of the training (blue) and testing (orange) losses, and the test metric (green) for PINN. (**d**) Experiment 16: History of the training (blue) and testing (orange) loss, and the test metric (green) for PINN algorithm. (**e**) Experiment 1: Residual error of $n\left(t\right)$ at randomly sampled 127 points. (**f**) Experiment 16: Residual error of $n\left(t\right)$ at randomly sampled 127 points.

Using five RTs, we performed 25 numerical experiments, in which the PINN algorithms were developed to predict $n\left(t\right)$ and ${c}_{i}\left(t\right)$ for six groups. As discussed in the Methods section, PINN was implemented using a fully connected feed forward neural network (FFNN). The list of Experiments indicating the training and testing domains in given in Tables 1 and 2 in the Discussion section. Examples of PINN performance (without transfer learning) are displayed in Fig. 2 for Experiments 1 and 16, which involve predictions of RT-1 and RT-4, respectively.

**Table 1:** Model structures, reactor transients, computational costs, MSEs, and relative errors.

Experiment	Model	Pre-train	Predicted transient	Iterations	Learning rate	Time [s]	Train loss (MSE)	Test loss (MSE)	Test metric (L_2_)
1	PINN	–	RT-1	95,000	0.0003	62.94	6.58·10^–5^	8.08·10^–5^	1.19·10^–4^
2	TL-PINN	RT-2	RT-1	4000	0.001	2.65	2.36·10^–4^	2.86·10^–4^	1.62·10^–4^
3	TL-PINN	RT-3	RT-1	7000	0.001	4.14	1.52·10^–4^	1.27·10^–4^	1.66·10^–4^
4	TL-PINN	RT-4	RT-1	9000	0.002	5.06	3.63·10^–4^	3.72·10^–4^	1.98·10^–4^
5	TL-PINN	RT-5	RT-1	10,000	0.001	5.48	2.91·10^–4^	2.83·10^–4^	2.64·10^–4^
6	PINN	–	RT-2	95,000	0.0003	63.37	8.15·10^–5^	8.81·10^–5^	2.50·10^–4^
7	TL-PINN	RT-1	RT-2	5000	0.0003	3.08	2.31·10^–4^	2.06·10^–4^	4.69·10^–4^
8	TL-PINN	RT-3	RT-2	3000	0.001	2.01	2.37·10^–4^	2.27·10^–4^	3.77·10^–4^
9	TL-PINN	RT-4	RT-2	4000	0.001	2.56	2.39·10^–4^	2.11·10^–4^	2.65·10^–4^
10	TL-PINN	RT-5	RT-2	6000	0.0003	3.63	1.89·10^–4^	2.02·10^–4^	4.36·10^–4^
11	PINN	–	RT-3	93,000	0.0003	68.22	8.04·10^–5^	8.76·10^–5^	3.22·10^–4^
12	TL-PINN	RT-1	RT-3	12,000	0.002	6.62	2.57·10^–4^	2.55·10^–4^	4.24·10^–4^
13	TL-PINN	RT-2	RT-3	9000	0.0001	5.37	3.96·10^–4^	4.42·10^–4^	4.52·10^–4^
14	TL-PINN	RT-4	RT-3	3000	0.0006	2.03	6.45·10^–5^	7.45·10^–5^	2.74·10^–4^
15	TL-PINN	RT-5	RT-3	3000	0.001	2.06	4.89·10^–4^	6.01·10^–4^	2.82·10^–4^
16	PINN	–	RT-4	95,000	0.0003	68.36	5.47·10^–5^	8.89·10^–5^	2.76·10^–4^
17	TL-PINN	RT-1	RT-4	18,000	0.001	9.83	2.05·10^–4^	2.07·10^–4^	4.74·10^–4^
18	TL-PINN	RT-2	RT-4	13,000	0.0001	7.48	3.55·10^–4^	4.85·10^–4^	5.11·10^–4^
19	TL-PINN	RT-3	RT-4	0	–	0.19	8.04·10^–5^	8.76·10^–5^	3.22·10^–4^
20	TL-PINN	RT-5	RT-4	6000	0.0003	3.69	3.39·10^–4^	4.32·10^–4^	2.94·10^–4^
21	PINN	–	RT-5	105,000	0.0003	72.63	1.04·10^–4^	1.11·10^–4^	4.64·10^–4^
22	TL-PINN	RT-1	RT-5	14,000	0.0003	7.84	1.29·10^–4^	1.40·10^–4^	4.86·10^–4^
23	TL-PINN	RT-2	RT-5	15,000	0.0001	8.53	2.06·10^–4^	2.49·10^–4^	4.99·10^–4^
24	TL-PINN	RT-3	RT-5	15,000	0.0001	8.38	5.84·10^–5^	6.04·10^–5^	5.01·10^–4^
25	TL-PINN	RT-4	RT-5	7000	0.0003	4.03	8.21·10^–5^	7.95·10^–5^	4.12·10^–4^

The total number of iterations in an operational sequence can be obtained by adding the corresponding number of iterations for PINN and TL-PINN models.

**Table 2:** Mean residual errors of neutron and precursors density for PINN and TL-PINN predictions of RT-1, RT-2, RT-3, RT-4, and RT-5.

Experiment	Model	Pre-train	Predicted transient	Mean residual error [%]						
				*n*	*c*_1_	*c*_2_	*c*_3_	*c*_4_	*c*_5_	*c*_6_
1	PINN	–	RT-1	0.3303	0.0029	0.0054	0.0166	0.0418	0.0887	0.5519
2	TL-PINN	RT-2	RT-1	0.4908	0.0049	0.0066	0.0270	0.0512	0.2166	0.3638
3	TL-PINN	RT-3	RT-1	0.4097	0.0035	0.0077	0.0210	0.0443	0.3189	0.5575
4	TL-PINN	RT-4	RT-1	0.8729	0.0051	0.0082	0.0336	0.6106	0.1432	2.0064
5	TL-PINN	RT-5	RT-1	0.7955	0.0072	0.0098	0.0272	0.0826	0.3615	2.1812
6	PINN	–	RT-2	0.4371	0.0045	0.0113	0.0360	0.0778	0.1577	0.3585
7	TL-PINN	RT-1	RT-2	0.6549	0.0102	0.0231	0.0665	0.1311	0.2545	0.7020
8	TL-PINN	RT-3	RT-2	0.5835	0.0073	0.0199	0.0602	0.1139	0.1735	0.5727
9	TL-PINN	RT-4	RT-2	0.5477	0.0075	0.0113	0.0328	0.0809	0.1965	1.0983
10	TL-PINN	RT-5	RT-2	0.8738	0.0219	0.0444	0.1263	0.2372	0.4429	0.4070
11	PINN	–	RT-3	0.5498	0.0071	0.0159	0.0445	0.0895	0.1673	0.3081
12	TL-PINN	RT-1	RT-3	0.6625	0.0091	0.0186	0.0577	0.1151	0.2337	1.4637
13	TL-PINN	RT-2	RT-3	0.5490	0.0132	0.0234	0.0641	0.1161	0.1982	0.8151
14	TL-PINN	RT-4	RT-3	0.5230	0.0057	0.0134	0.0413	0.0810	0.1613	0.3776
15	TL-PINN	RT-5	RT-3	0.5715	0.0077	0.1301	0.0418	0.0818	0.1839	0.3742
16	PINN	–	RT-4	0.5211	0.0061	0.0134	0.0378	0.0807	0.1596	0.2976
17	TL-PINN	RT-1	RT-4	0.6739	0.0078	0.0229	0.0659	0.1239	0.2336	0.9247
18	TL-PINN	RT-2	RT-4	0.5927	0.0160	0.0273	0.0724	0.1269	0.2177	0.7603
19	TL-PINN	RT-3	RT-4	0.5498	0.0071	0.0159	0.0446	0.0895	0.1673	0.3081
20	TL-PINN	RT-5	RT-4	0.5076	0.0080	0.0141	0.0391	0.0845	0.1614	0.4719
21	PINN	–	RT-5	0.6049	0.0094	0.0229	0.0648	0.1146	0.2197	0.4187
22	TL-PINN	RT-1	RT-5	0.6898	0.0010	0.0244	0.0637	0.1195	0.2219	0.4050
23	TL-PINN	RT-2	RT-5	0.5569	0.0093	0.0243	0.0661	0.1239	0.2361	0.5579
24	TL-PINN	RT-3	RT-5	0.6715	0.1179	0.0252	0.0699	0.1232	0.2186	0.4777
25	TL-PINN	RT-4	RT-5	0.5722	0.0079	0.0203	0.0568	0.1058	0.1967	0.5602

In Experiment 1, PINN algorithm required 95,000 iterations to converge, with training/prediction wall time of 62.94 s. Figure 2a display $n\left(t\right)$ along with PINN training points and predictions. One can observe that PINN predictions closely follow the transients of $n\left(t\right)$ for the entire computational domain. Similar results, not shown here, were obtained for other six groups ${c}_{1}\left(t\right)$ through ${c}_{6}\left(t\right)$. Figure 2c displays the graphs of convergence of training and testing losses, and the test metric. The training and testing losses start at the value of 1.63 × 10^2^ and decrease to the values of 6.58 × 10^–5^ and 8.08 × 10^–5^, respectively. The test metric starts at the value of 1 and decreases to the value 1.19 × 10^–4^. The training and testing losses, and the test metric decrease by factors of 10^7^ and 10^6^, respectively, which suggests low errors for the PINN algorithm tracking of RT-1. Figure 2e shows the residual error for $n\left(t\right)$. The largest outlier error is approximately 6%, while the majority of errors are below 1%.

In Experiment 16, PINN algorithm required 95,000 iterations to converge (same as for Experiment 1), with the training/prediction wall time of 68.36 s (slightly larger than for Experiment 1). The results in Fig. 2b display $n(t)$ transient along with PINN training points and predictions. One can observe that PINN predictions closely follow $n(t)$ for the entire computational domain. Similar results, not shown here, were obtained for other six groups ${c}_{1}\left(t\right)$ through ${c}_{6}\left(t\right)$. Figure 2d displays evolution of losses during 95,000 iterations. The training and testing losses start at the value of 1.85 × 10^2^, and decrease to the values of 5.47 × 10^–5^ and 8.89 × 10^–5^, respectively. The test metric starts at the value of 1 and decreases to the value of 2.67 × 10^–4^. The training and testing losses, and the test metric decrease by a factor of 10^7^ and 10^6^, respectively, which suggests low performance errors of the PINN algorithm. Figure 2f shows the residual error for $n(t)$. The majority of the errors is below 1%. The largest error appears to be an outlier, with the value of slightly above 6%, which occurs for prediction at the start of the transient.

**Figure 3:**
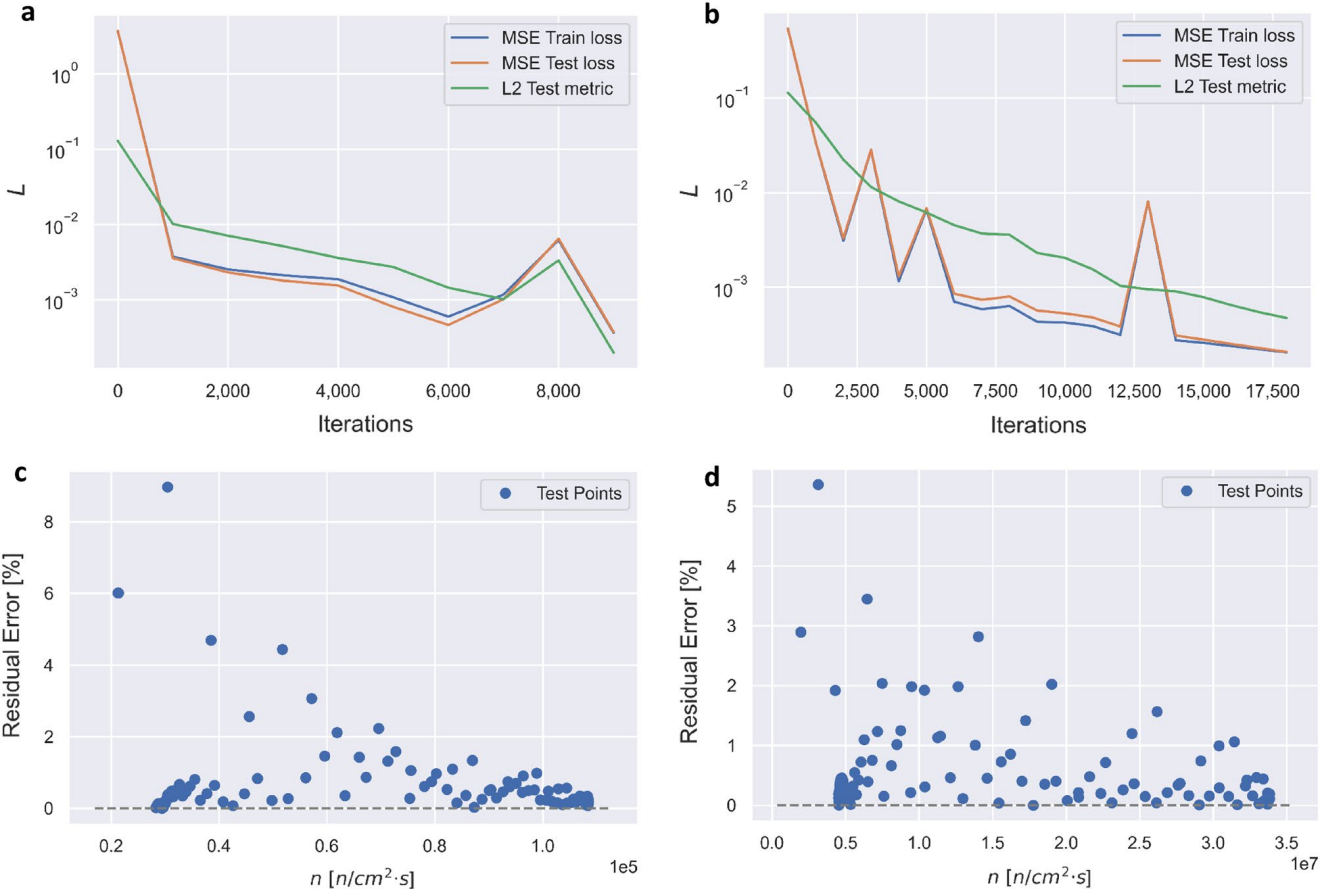
(**a**) Experiment 4: History of the loss function of TL-PINN for training (blue) and testing (orange) loss, and the test metric (green). (**b**) Experiment 17: History of the loss function of TL-PINN for training (blue) and testing (orange) loss, and test metric (green). (**c**) Experiment 4: Residual error plot of $n\left(t\right)$ randomly sampled at 127 points. (**d**) Experiment 17: Residual error plot of $n\left(t\right)$ at randomly sampled 127 points.

Examples of TL-PINN performance for Experiment 4 where RT-1 was predicted with the TL-PINN model pre-trained on RT-4, and Experiment 17 where RT-4 was predicted with TL-PINN model pre-trained on RT-1, are displayed in Fig. 3. In Experiment 4, we obtained similar results for predictions of $n(t)$ as those shown in Fig. 2a. In contrast to Experiment 1, the TL-PINN algorithm required 9000 iterations to converge in training on RT-1, with the training/prediction wall time of 5.06 s. Figure 3a displays the history of loss for up to 9000 iterations. The train and test loss have initial values of 3.68 and decrease to 1.65 × 10^–4^ and 1.64 × 10^–4^, respectively. The test metric starts at the value of 1.29 × 10^–1^ and decreases to 2.25 × 10^–4^. The train and test loss, and test metric decrease by factor of 10^4^ to 10^3^, respectively, which gives an expectation of low performance errors of PINN algorithm. Figure 3c shows the residual percentage error plot of the neutron density concentration $n\left(t\right)$. The largest outlier error is approximately 8.5%, but the majority of the errors are below 1%.

In Experiment 17, we obtained similar results for predictions of $n(t)$ and ${c}_{i}\left(t\right)$ as those shown in Fig. 2a and b. In contrast to Experiment 16, the TL-PINN algorithm required 18,000 iterations to converge in training on RT-4, with the training/prediction wall time of 9.83 s. Figure 3b displays the history of loss after 18,000 iterations. The train and test loss start at the value of 5.35 × 10^–1^ and decreases to 2.05 × 10^–4^ and 2.07 × 10^–4^, respectively. The test metric starts at 1.14 × 10^–1^ and decreases to 4.74 × 10^–4^. The train and test losses, and test metric decrease by a factor of 10^3^, which gives an expectation of low errors in PINN prediction. Figure 3d displays the residual percentage error plot of $n(t)$, randomly sampled at 127 points. The largest outlier error of approximately 5.5%, but the majority of errors are below 1%.

## Discussion

In this paper, we have demonstrated that TL-PINN, allows for significant acceleration of model training and testing. Five different RTs were generated via ODE45 solution of PKEs with coefficients taken from PUR-1. The transients, RT-1, RT-2, RT-3, RT-4, and RT-5, consist of reactor response to positive reactivity insertion. Summary of results of numerical experiments is presented in Table 1. The hypothetical sequence of operations involves two reactor transients. The first transient is predicted with PINN, and the second transient is predicted with TL-PINN. Acceleration of performance is achieved when using TL-PINN to predict the second transient instead of a PINN. Numerical experiments 1, 6, 11, 16, and 21 present the results for the first transient prediction with PINN, which provide the baseline performance metrics for TL-PINN performance evaluation. Numerical experiments in which the second transient is predicted with TL-PINN are grouped by the predicted RT. The 5th column of Table 1 lists the number of iterations for convergence. The learning rate for PINN models to reach convergence was determined to be 0.0003, while the learning rate for TL-PINN models in the 6th column of Table 1 ranges from 0.0001 to 0.002. Columns 7, 8, 9, and 10 list the values of model runtimes (wall clock), train loss calculated as MSE, test loss calculated as MSE, and test metric calculated as L_2_, respectively.

According to the results in Table 1, the pre-trained TL-PINN models require fewer iterations for convergence compared to conventional PINN models. In Experiments 2 to 5, TL-PINN models predicting RT-1 converge after 4000, 7000, 9000, and 10,000 iterations, respectively, as compared to 95,000 iterations for a PINN model in Experiment 1. This represents a nearly tenfold to 24-fold acceleration in prediction of RT-1 if another transient was previously learned. The training/testing time for RT-1 prediction decreases from approximately 63 s with PINN to approximately 2–6 s with TL-PINN, which places TL-PINN performance in the range of near real-time operation.

In Experiments 7 to 10, TL-PINN models predicting RT-2 converge after 3000, 4000, 5000, and 6000 iterations, respectively, as compared to 95,000 iterations for a PINN model in Experiment 6. This represents a nearly 16-fold to 32-fold acceleration for prediction of RT-2 if another transient was previously learned. The training/testing time for prediction of RT-2 decreases from approximately 64 s with PINN to approximately 2–4 s with TL-PINN, which places TL-PINN performance in the range of near real-time operation.

Similar performance acceleration is achieved for TL-PINN models predicting RT-3, RT-4, and RT-5. For almost all RTs, the train losses, test losses, and test metrics are approximately an order of magnitude smaller for conventional PINNs relative to TL-PINNs, mainly due to the different convergence criteria. Experiments 14, 19, 22, 23, 24, and 25 that utilize TL-PINN models, achieve similar or lower train losses, test losses, and test metrics compared to conventional PINN models.

As an example, we consider an operational sequence consisting of the transient RT-1 followed by the transient RT-2. Using PINNs only (Experiments #1 and #6 in Table 1) would require 95,000 iterations for predicting each transient, for a total of 190,000 iterations in this operational scenario. On the other hand, using TL-PINN for RT-2 pre-trained on RT-1 (Experiment #7 in Table 1) would involve 5000 iterations, reducing the total number of iterations in this operational scenario to 100,000.

Table 2 lists the mean residual errors for PINN and TL-PINN predictions of 25 neutron and 150 neutron precursor densities for RT-1, RT-2, RT-3, RT-4, and RT-5. For all five RTs, the mean residual errors for PINN and TL-PINN predictions are of the same order of magnitude for respective variables.

The general trend is that for all cases, the average for $n\left(t\right)$ is slightly larger for predictions with TL-PINN. However, the average errors in prediction of $n\left(t\right)$ for all 25 experiments in this study are below 1%, which is sufficient accuracy for reactor monitoring. The average errors in prediction of n (t) with PINN are in the range 0.33 to 0.61%, and the errors in predictions of $n\left(t\right)$ with TL-PINN are in the range 0.41 to 0.87%. The errors for 146 neutron precursor densities range from 0.0029 to 0.92%. The exceptions are outliers for TL-PINN predictions of four neutron precursor densities ${c}_{6}$ with mean errors of 1.09%, 1.46%, 2%, and 2.18%. Unlike neutron density, the precursor density is not experimentally measurable. The average residual errors in predictions of ${c}_{i}\left(t\right)$ are generally smaller than those of $n\left(t\right)$. The general trend for all five RTs is that the errors for ${c}_{i}\left(t\right)$ are larger for predictions with TL-PINNs compared to those of conventional PINNs.

To elucidate the criteria for performance of transfer learning for different domains, we calculated the Hausdorff distance similarity measure between different RTs. Table 3 lists the values of Hausdorff distance similarity measure between scaled RTs, as shown in Fig. 1b. Hausdorff distance is symmetric, with zero value indicating maximum similarity between two curves. According to the values of Hausdorff distance, RT-1 is most similar to RT-2, with similarity progressively decreasing RT-3 and RT-4, and RT-5. For RT-2, the order of transients in decreasing similarity is RT-3, RT-1, RT-4 and RT-5. RT-3 is most similar with RT-4. RT-5 is most similar to RT-3, but shows the largest Hausdorff distance value among all RTs.

**Table 3:** Hausdorff distance similarity measure between RT-1, RT-2, RT-3, RT-4, and RT-5.

RT	RT-1	RT-2	RT-3	RT-4	RT-5
RT-1	0	0.105	0.202	0.232	0.272
RT-2	0.105	0	0.097	0.127	0.174
RT-3	0.202	0.097	0	0.038	0.138
RT-4	0.232	0.127	0.038	0	0.161
RT-5	0.272	0.174	0.138	0.161	0

**Figure 4:**
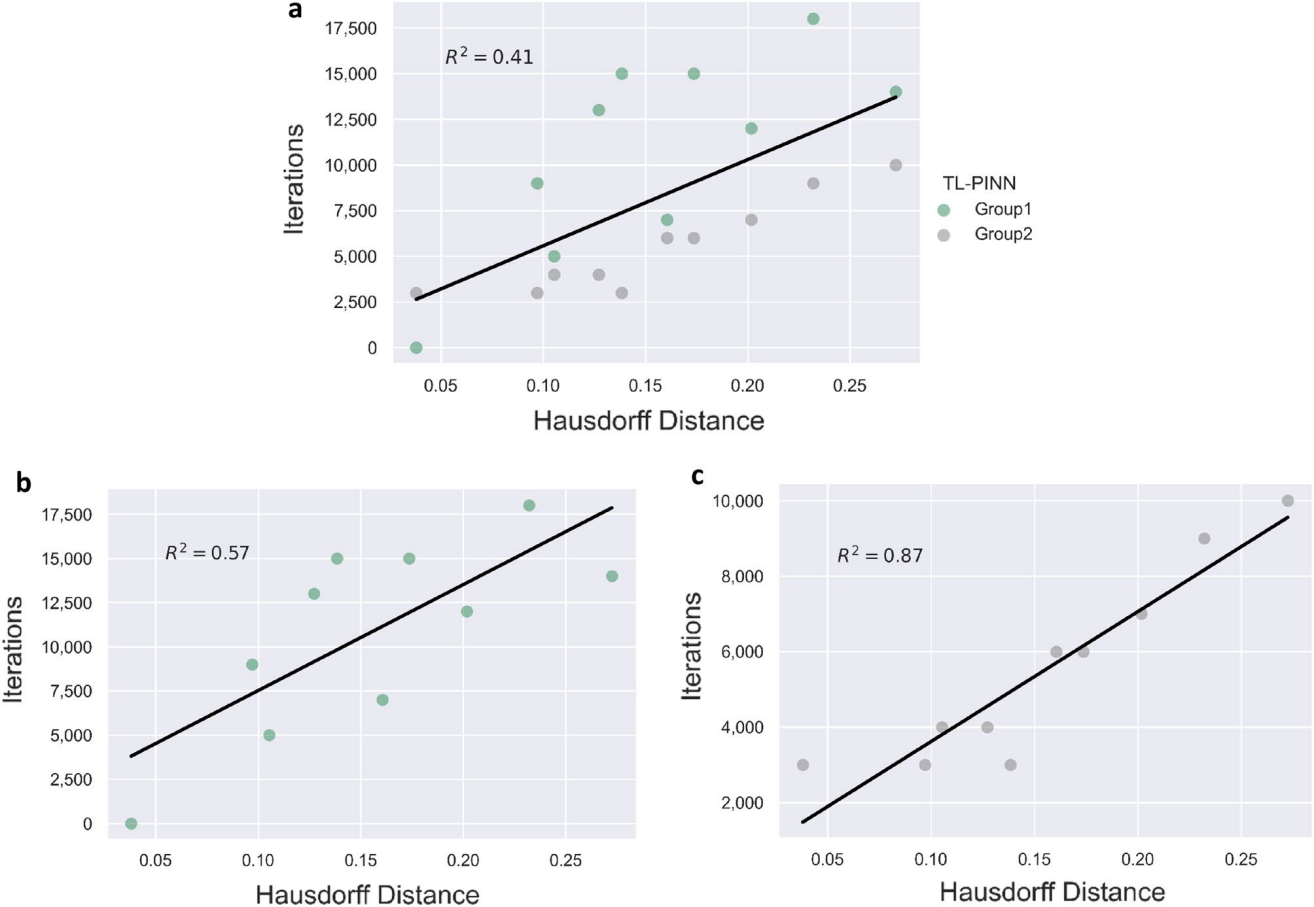
(**a**) Number of iterations for convergence vs. Hausdorff distance. Group 1: TL-PINNs were pre-trained on RTs with lower respective amplitudes than those of the predicted RTs. Group 2: TL-PINNs were pre-trained on RTs with larger respective amplitudes compared to the predicted RTs. The linear regression fit is ${R}^{2}=0.41$. (**b**) Number of iterations for convergence vs. Hausdorff distance for the TL-PINN for Group 1. The best linear fit has ${R}^{2}=0.57$. (**c**) Number of iterations for convergence vs. Hausdorff distance for Group 2. The best linear fit has ${R}^{2}=0.87$.

Figure 4a shows the number of iterations for TL-PINNs’ convergence as a function of the Hausdorff distance. The data points were divided into two groups to highlight the following trend. Group 1 includes the results where TL-PINN was pre-trained on RTs with lower respective amplitudes than those of the predicted RTs. Group 2 includes the cases where TL-PINN algorithms were pre-trained on RTs with larger respective amplitudes than those of the predicted RTs. TL-PINNs of Group 1 needed more iterations to converge compared to TL-PINNs of Group 2 for all experiments except experiment 19. In addition, the statistical measure ${R}^{2}$ of the linear regression was calculated. The best linear fit to data in Fig. 4a has the value of ${R}^{2}=0.41$. The relation between Hausdorff distance and transfer learning performance does not appear to be linear, but the general trend is that smaller Hausdorff distances between RTs correlates with better transfer learning performance.

To further demonstrate the trends, the data for Groups 1 and 2 in Fig. 4a was decomposed into Fig. 4b and c, respectively. Figure 4b indicates that as Hausdorff distance value becomes larger, the number of iterations needed for TL-PINN algorithms to converge fluctuates to larger values. More specifically, the relation between Hausdorff distance and transfer learning performance does not seem to be linear, as the iterations needed for convergence gradually increase but decline in four cases to lower values when the Hausdorff distance is equal to 0.105, 0.161, 0.202, and 0.272. However, a pattern shows that usually when Hausdorff distance is shorter, better transfer learning performance is achieved. The plotted line shows the best linear fit to data and has the value of ${R}^{2}=0.57$. Figure 4c shows that as Hausdorff distance value becomes larger, the number of iterations needed for TL-PINN algorithms to converge increases or remain the same for every experiment tested, except experiment 15. The relation between Hausdorff distance and transfer learning performance appears to be linear. The strong relation is captured by the plotted best linear fit line that has the value of ${R}^{2}=0.87$.

In the numerical experiments conducted in this study it was found that the best TL-PINN performance was obtained when using the smallest Hausdorff distance pre-trained on RTs with smaller maximum amplitude than the predicted RTs. According to these results, in a real-world scenario, the reactor operator should be able to identify the most suitable pre trained TL-PINN algorithm for a target RT, using the Hausdorff distance metric and the maximum amplitude of target RT.

Furthermore, TL-PINN algorithms presented low computational time (ranging from 0.2 to 10 s) that can offer near real-time operation using conventional hardware. Real-world nuclear applications that leverage higher-level hardware, can potentially minimize the TL-PINN runtime from seconds to milliseconds, achieving real-time prediction. In the future work, we will investigate prediction of experimental PUR-1 transients using TL-PINN model pre-trained using computer simulations of different transients. We anticipate that the error in TL-PINN predictions will depend on the degree of agreement between the DE model and the experimental observations.

## Methods

### Point kinetics equations (PKEs)

The PKEs consist of a system of stiff nonlinear coupled differential equations, which model the kinetics of reactor variables, including neutron density concentration, the delayed neutron precursor density concentration for six groups, and reactivity. PKEs are the reduced order model of the Boltzmann neutron transport equation and the Bateman equation describing 3D spatial and temporal kinetics of a nuclear reactor. The PKEs are derived under the approximation that both the shape of the neutron flux and the neutron density distribution are ignored, thus assuming that the reactor acts as a point^30^. This approximation is generally valid for small reactors under the condition of relatively small reactivity insertion. Solution of PKEs provides information of the nuclear reactor power level and the power fluctuation during reactivity transient. The PKEs for *i* groups of delayed neutrons are given as^30^:

1 $$\frac{{dn}\left({t}\right)}{{dt}}=\frac{\uprho \left({t}\right)-\upbeta }{\Lambda }\cdot {n}\left({t}\right)+\sum_{{i}}{\uplambda }_{{i}}\cdot {{c}}_{{i}}\left({t}\right),$$

2 $$\frac{{{dc}}_{{i}}\left({t}\right)}{{dt}}=\frac{{\upbeta }_{{i}}}{\Lambda }\cdot {n}\left({t}\right)-{\uplambda }_{{i}}\cdot {{c}}_{{i}}\left({t}\right),$$

where, $n$ represents the neutron density concentration, ${c}_{i}$ is the delayed neutron precursor density concentration for group $i$, $\rho$ is the reactivity feedback which is function of time $t$, ${\beta }_{i}$ is the delayed neutron fraction for each group, $\beta =\sum {\beta }_{i}$ is the sum of the delayed neutron fractions. In addition, $\Lambda$ is the mean neutron lifetime in the reactor core, and ${\lambda }_{i}$ is the mean neutron precursor lifetime for each group $i$. At time $t=0$, the reactor is in steady state, and we use the following initial conditions^30^:

3 $${n}\left(0\right)={{n}}_{0},$$

4 $${{c}}_{{i}}\left(0\right)=\frac{{\upbeta }_{{i}}}{{\uplambda }_{\upiota }\cdot\Lambda }\cdot {{n}}_{0},$$

where the values of ${\beta }_{i}$, ${\lambda }_{i}$, and $\Lambda$ are suggested by the experimental data. In most systems, ${\beta }_{i}/\left({\lambda }_{i}\cdot \Lambda \right)\gg 1$*,* and therefore under steady state conditions we obtain ${c}_{i}\gg n$^30^. Because of stiffness of the ODE system, numerical solution of the PKEs requires using relatively small time steps in the computational domain to achieve accurate solution.

Reactivity $\rho$ defined as the deviation of an effective multiplication factor ${k}_{eff}$ from unity, is a measure of the state of a reactor relative to critical state^30^. When $\rho <0$ the reactor is subcritical, when $\rho =0$ the reactor is critical, and when $\rho >0$ the reactor is supercritical. Reactivity is a dimensionless number, but it is commonly expressed in per cent mile or pcm units.

In this work, we generate five RTs (RT-1, RT-2, RT-3, RT-4, and RT-5), which are responses to five different reactivity insertion schedules. All reactivity curves start with positive values, with the range of initial values between 500 and 600 pcm. The reactivity curves remain constant for approximately the first 200 s, gradually decreases to zero value between 200 and 500 s, and remains at zero until the end of the transient at 742 s. In developing reactivity insertion schedules, the objective was to obtain five different neutron density transients with values in the range of typical experimental observations for PUR-1 operation. Because Python library computations are performed on normalized time domain $t\in \left[\mathrm{0,1}\right]$ s, as discussed in the Methods section, the reactivity values were scaled to be two orders of magnitude higher than typical values of PUR-1. Table 4 lists the reactivity schedule equations in the normalized time domain, and neutron density initial conditions for the five reactor transients.

**Table 4:** Reactivity schedule equations in the normalized time domain and neutron density initial values.

Transient	Reactivity schedule [*pcm*]	Neutron density initial values [*n/cm*^2^*·s*]
RT-1	${\rho }_{1}\left(t\right)=500{e}^{-20(t-0.49)}/\left(1+{e}^{-20(t-0.49)}\right)$	${n}_{1}\left(0\right)=0.2\cdot {10}^{5}$
RT-2	${\rho }_{2}\left(t\right)=570{e}^{-14(t-0.55)}/\left(1+{e}^{-14(t-0.55)}\right)$	${n}_{2}\left(0\right)=0.2\cdot {10}^{6}$
RT-3	${\rho }_{3}\left(t\right)=590{e}^{-14(t-0.51)}/\left(1+{e}^{-20(t-0.51)}\right)$	${n}_{3}\left(0\right)=0.2\cdot {10}^{6}$
RT-4	${\rho }_{4}\left(t\right)=600{e}^{-20(t-0.49)}/\left(1+{e}^{-20(t-0.49)}\right)$	${n}_{4}\left(0\right)=0.2\cdot {10}^{7}$
RT-5	${\rho }_{5}\left(t\right)=600{e}^{-14(t-0.59)}/\left(1+{e}^{-14(t-0.59)}\right)$	${n}_{5}\left(0\right)=0.2\cdot {10}^{8}$

### Purdue university reactor number one (PUR-1)

**Figure 5:**
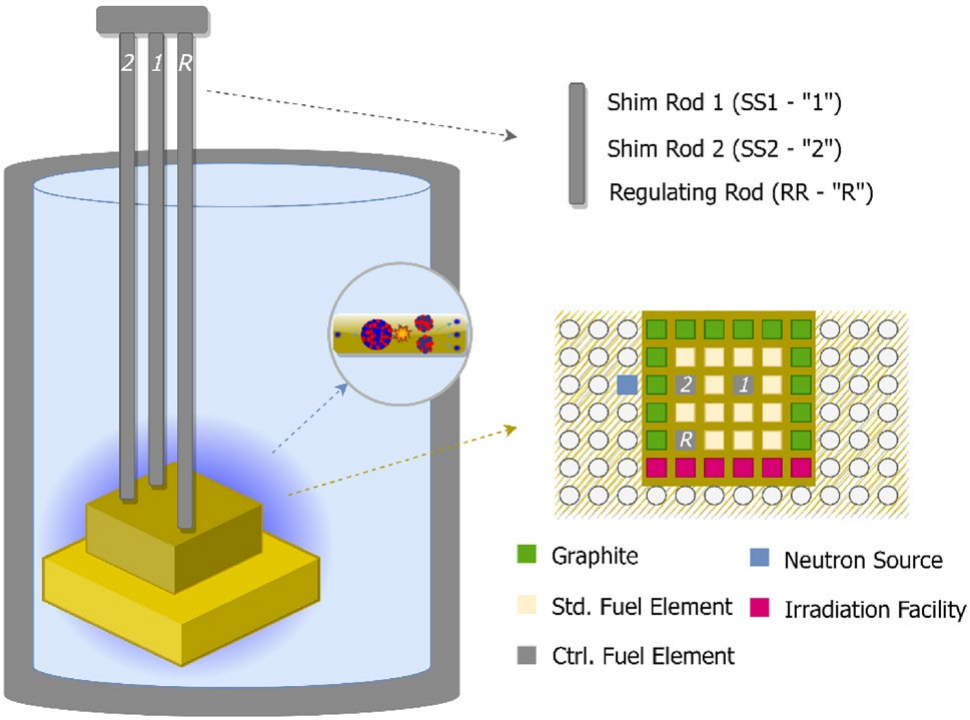
Schematics of PUR-1.

PUR-1 is an all-digital 10kWth material test reactor (MTR) – pool type, with flat plate type fuel by BWXT Technologies^31^. Fuel material consists of high essay low enriched uranium (19.75% ^235^U) in the form of U_3_Si_2_ – Al. There are 16 total assemblies, where each standard assembly has up to 14 fuel elements. The core is submerged into a 5.2 m deep water pool, where water is used for both neutron moderation and fuel heat removal. Average thermal neutron flux in the fuel region is 1.2 × 10^10^ n/cm^2^·s, with the maximum thermal flux reaching the value of 2.1 × 10^11^ n/cm^2^·s. The reactor power is controlled with three control rods. Two of them are borated stainless steel shim safety rods (SS1 and SS2), and the third one is 304 stainless steel regulating rod (RR). Figure 5 shows a schematic drawing of PUR-1 and an inset panel with the relative locations of the fuel elements and control rods. In principle, correlations can be established between reactivity and position of control rods^21^.

The PKEs with six groups of delayed neutron precursors were constructed using coefficients obtained from MCNP simulations of PUR-1. The values of ${\beta }_{i}$ and ${\lambda }_{i}$ are listed in Table 5, while $\Lambda =1.2\cdot {10}^{10} s$.

**Table 5:** Parameters of PUR-1 for the PKE model development.

Variable	Value [s]					
Term	1	2	3	4	5	6
${\beta }_{i}$	0.000213	0.001413	0.001264	0.002548	0.000742	0.000271
${\lambda }_{i}$	0.01244	0.0305	0.1114	0.3013	1.1361	3.013

### Physics-informed neural network (PINN) architecture

**Figure 6:**
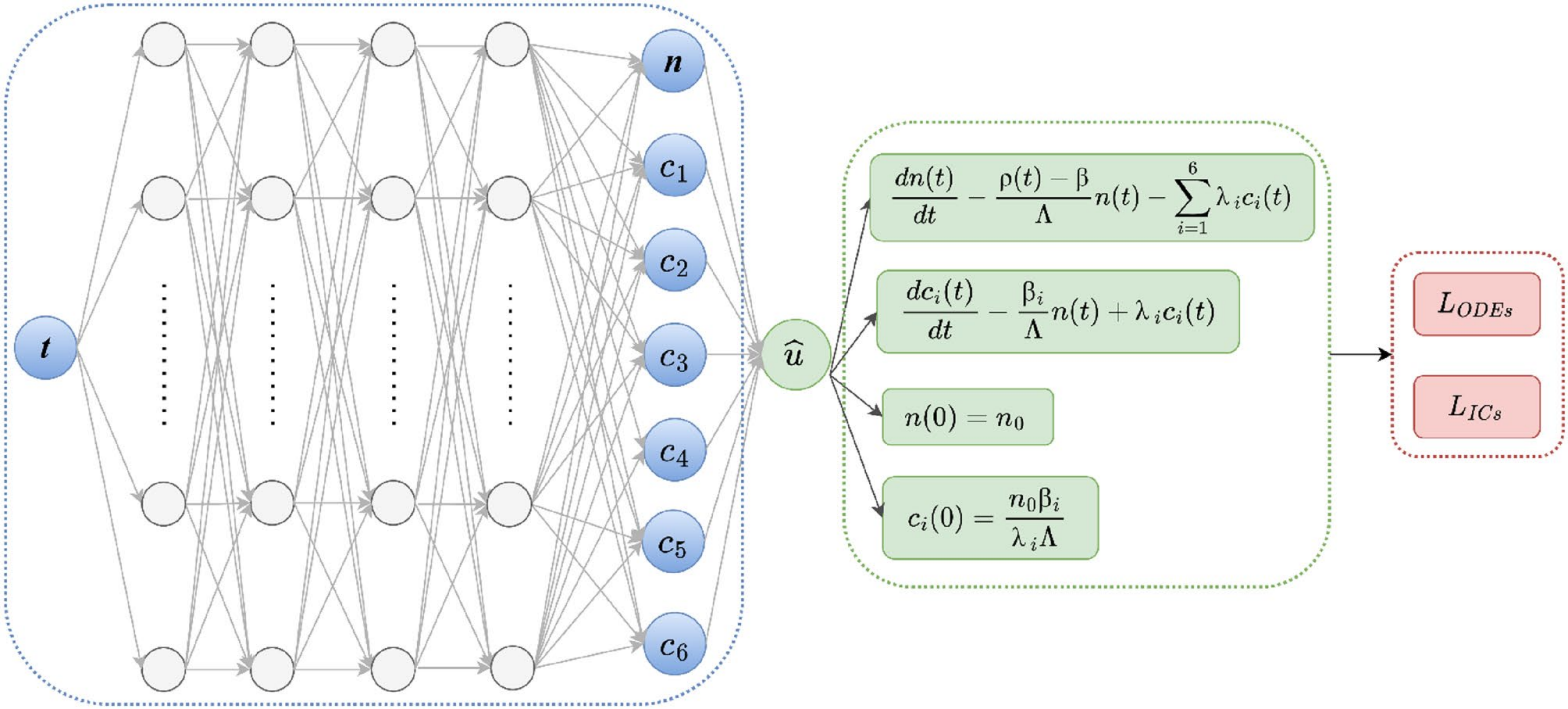
Schematic of PINN for solving the PKEs with ICs. The input to surrogate network is time t, and the output is the solution vector ${\left[n\left(t\right), {c}_{1}\left(t\right), {c}_{2}\left(t\right), {c}_{3}\left(t\right),{ c}_{4}\left(t\right),{ c}_{5}\left(t\right), {c}_{6}\left(t\right)\right]}^{T}$. The residual network tests if the solution vector satisfies the PKE governing equations and the ICs.

The architecture of PINN algorithm developed in this paper is shown in Fig. 6. The PINN is consisted of the surrogate and the residual network. The input of the surrogate network is a point in the computational time domain, also called the collocation point. A feed-forward neural network (FFNN) delivers the PKEs approximated solution, that is the state vector ${\left[n\left(t\right), {c}_{1}\left(t\right), { c}_{2}\left(t\right), { c}_{3}\left(t\right), { c}_{4}\left(t\right), { c}_{5}\left(t\right), {c}_{6}\left(t\right)\right]}^{T}$. The weights of the surrogate network are trainable. The input to the residual network is the output of the surrogate network. The residual network includes the governing PKEs and the ICs, and calculates the residual that is used as a loss function to optimize the surrogate network. The loss function $\mathcal{L}$ is defined by the sum of the mean squared residual of the governing equations and initial conditions. The total loss is written as:

5 $$\mathcal{L}\left(\theta ;T\right)={w}_{f}{\mathcal{L}}_{f}\left(\theta ;{T}_{f}\right)+{w}_{i}{\mathcal{L}}_{i}\left(\theta ;{T}_{i}\right),$$

where ${w}_{f}$ and ${w}_{i}$ are the scalar weights for the ODEs and the ICs, respectively. The scalar weights keep the loss terms balanced at the start of the training, so each term contributes equally. The FFNN’s weights and biases are the parameter *θ*. The variables ${T}_{f}$ and ${T}_{i}$ are the training points inside the domain and at the IC’s. These sets of points are referred to as the residual points. The terms ${\mathcal{L}}_{f}$ and ${\mathcal{L}}_{i}$ are the mean squared error (MSE) of the residuals for the ODEs and ICs, respectively:

6 $${\mathcal{L}}_{f}\left(\theta ;{T}_{f}\right)=\frac{1}{\left|{T}_{f}\right|}\sum_{x\epsilon {T}_{f}}{\left\Vert f\left(x; \frac{d\widehat{\mathrm{u}}}{dx}\right)\right\Vert }_{2}^{2},$$

7 $${\mathcal{L}}_{i}\left(\theta ;{T}_{i}\right)=\frac{1}{\left|{T}_{i}\right|}\sum_{x\epsilon {T}_{i}}{\Vert I\left(\widehat{\mathrm{u}}, x\right)\Vert }_{2}^{2},$$

where $\widehat{u}$ is the approximate numerical solution obtained from the surrogate network. The derivatives in the loss function are calculated using automatic differentiation.

In this work, PINN implementation is based on FFNN, consisting of four-layers with hyperbolic tangent (*tanh*) as the activation function. The same activation function is applied to all layers, except for the output layer, which does not use any activation function. The FFNN input layer comprises of a single input variable, which is a point in the time domain. The hidden layers consist of 32 neurons. The output layer produces the final prediction of seven quantities, which are the neutron density concentration $n$ and the delayed neutron precursor’s density concentration *c*_*i*_ for six groups. The Xavier Glorot method is used to initialize the weights of the FFNN, which is the most suitable initialization method when *tanh* is the activation function^32^. The Adam optimizer with a learning rate of $\lambda =0.0003$ is used to minimize the loss function. The training data set is obtained using the ODE45 solver, and consists of 32 collocation points. The training data are divided into 30 training points inside the solution domain, and two training points at the ICs. The collocation points are distributed according to the Sobol sequence. The testing data consists of randomly distributed 127 points. The residuals are evaluated in the computational domain of the reactor transient time $t\in [0, 742]s$. Implementation of PINN DeepXDE^33^ library utilized in this paper requires feature scaling, which is a common procedure in machine learning. Therefore, the time domain was normalized to be in the range [0, 1], and the neutron density concentration $n(t)$ was scaled to the range [0, 1]. Delayed neutron precursors density concentrations ${c}_{i}(t)$ were scaled correspondingly. After training, PINN predictions were scaled up to the original “physical” range of values.

Transfer learning with PINNs was performed by storing the matrix of coefficients from training/testing on one transient, and then using these values as initial guesses for training/testing on another transient. Table 6 describes the sequence of steps of PINN and TL-PINN algorithm.

**Table 6:** Sequence of steps in PINN and TL-PINN algorithms.

Step #	PINN procedure
Step 1	Specify the computational domain
Step 2	Specify the system of ODEs
Step 3	Specify the training data, their distribution, and volume
Step 4	Construct a FFNN
Step 5	Define a model by combining the system of ODEs and the FFNN
Step 6	Set the optimization hyperparameters
Step 7	Train the network using specified initialization
Step 8	Predict the ODEs solution
Step 9	Save the model

–	TL-PINN procedure
Step 10	Repeat steps 1–6
Step 11	Restore model from Step 9
Step 12	Train the network using restored model’s initialization
Step 13	Predict the ODEs solution

All calculations were performed on Windows PC with AMD Ryzen 7 5800H with Radeon Graphics, 8 cores processor, and 32 GB of RAM. The training and testing losses are calculated as the mean squared error (MSE) for $n\left(t\right)$ and six ${c}_{i}\left(t\right)$. We chose to calculate the test metric as ${\mathcal{L}}_{2}$ relative error of $n\left(t\right)$ because neutron density can be measured experimentally. The data for test metric consists of randomly distributed 127 points. The convergence criterion for conventional PINNs and TL-PINNs was set as follows. PINN algorithms were trained until training loss, testing loss, and test metric reached simultaneously the range of 10^–5^, 10^–5^, and 5·10^–4^ respectively, and up to maximum of 105,000 iterations. TL-PINN algorithms were trained until training loss, testing loss, and test metric reached simultaneously the range of 10^–4^, 10^–4^, and 5·10^–4^ respectively. This criterion ensured that the mean error of $n\left(t\right)$ stays below 1%. Conventional PINNs convergence criterion is stricter than TL-PINNs because pre training in more accurate solutions facilitates faster convergence of TL-PINN algorithms.

### Similarity measures

To elucidate the criteria for performance of transfer learning for different domains, this study investigated several similarity measures between reactor transient curves. The metrics include partial curve mapping (PCM), Fréchet distance, area between two curves, dynamic time warping distance, and Hausdorff distance. Different similarity metrics indicated to the same relationship between reactor transient curves, with Fréchet distances numerically equal to Hausdorff distance for all transients. Therefore, in this paper we limit the presentation to Hausdorff distance^34^ similarity measure.

The Hausdorff distance is a measure of dissimilarity between two point sets. The directed Hausdorff distance $\check{H}$ between two point sets $A$ and $B$ is not symmetric, and gives the maximum of distances between each point $x\in A$ and its nearest neighbor $y\in B$. The directed Hausdorff distance is given as:

8 $$\check{H}\left(A, B\right)={max}_{x\in A}\left\{{min}_{y\in B}\{\Vert x, y\Vert \}\right\},$$

where $\Vert x, y\Vert$ is the Euclidean distance function. The Hausdorff distance $H$ is the maximum of the directed Hausdorff distances in both directions, and thus it is symmetric. The Hausdorff distance is given as:

9 $$H\left(A, B\right)=max\left\{\check{H}\left(A, B\right), \check{H}\left(B, A\right)\right\}.$$

## Data Availability

The datasets used and/or analyzed during the current study available from the corresponding author on reasonable request.

## Code availability

The codes used and/or analyzed during the current study available from the corresponding author on reasonable request.
